# Total body irradiation with a reconditioned cobalt teletherapy unit

**DOI:** 10.1120/jacmp.v7i1.2175

**Published:** 2006-02-21

**Authors:** Michael D.C. Evans, Renée‐Xavière Larouche, Marina Olivares, Pierre Léger, Joe Larkin, Carolyn R. Freeman, Ervin B. Podgorsak

**Affiliations:** ^1^ Department of Medical Physics McGill University Health Centre 1650 ave. Cedar Montreal QC H3G 1A4 Canada; ^2^ Department of Radiation Oncology McGill University Health Centre 1650 ave. Cedar Montreal QC H3G 1A4 Canada

**Keywords:** total body irradiation, cobalt teletherapy

## Abstract

While the current trend in radiotherapy is to replace cobalt teletherapy units with more versatile and technologically advanced linear accelerators, there remain some useful applications for older cobalt units. The expansion of our radiotherapy department involved the decommissioning of an isocentric cobalt teletherapy unit and the replacement of a column‐mounted 4‐MV LINAC that has been used for total body irradiation (TBI). To continue offering TBI treatments, we converted the decommissioned cobalt unit into a dedicated fixed‐field total body irradiator and installed it in an existing medium‐energy LINAC bunker. This article describes the logistical and dosimetric aspects of bringing a reconditioned cobalt teletherapy unit into clinical service as a total body irradiator.

PACS numbers: 87.53.Dq, 87.53.Mr

## I. INTRODUCTION

The recent expansion and upgrading of our radiotherapy department involved the decommissioning of a 30‐year‐old isocentric cobalt teletherapy unit (Theratron‐780, AECL, Ottawa, Canada) and the replacement of a column‐mounted 4‐MV LINAC (Therapi 4, SHM, Sunnyvale, CA), which has been used for total body irradiation (TBI) with a sweeping beam technique^(^ ^1^ ^,^ ^2^ ^)^ for 25 years. To continue offering TBI treatments, we converted the decommissioned cobalt unit into a dedicated total body irradiator and installed it in an existing medium‐energy LINAC bunker. The bunker now houses two treatment units: the cobalt unit dedicated for use in TBI and a medium‐energy 10‐MV LINAC (Clinac 18, Varian, Palo Alto, CA) used for routine treatments as well as for special procedures, such as stereotactic radiosurgery and total skin electron irradiation.

## II. METHODS

During departmental renovations, the head‐and‐neck assembly of the cobalt unit was temporarily removed from the gantry ring of the main frame, and the cobalt source was locked in the fully shielded position located in the head. The piston mechanism, used to move the source drawer from the shielded to the unshielded or “beam‐on” position, was removed and stored. The inter‐leaved depleted uranium collimator was removed, the head‐and‐neck assembly was placed in a storage container, and the main frame of the teletherapy unit was scrapped.

**Figure 1:**
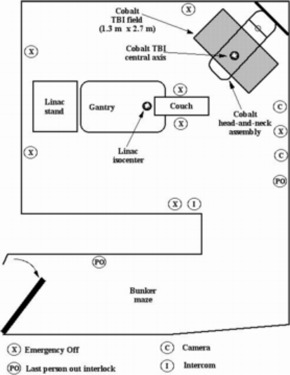
A floor plan of the treatment bunker and maze showing the location of the 10‐MV LINAC isocenter and the location of the field size and central axis of the cobalt unit. The safety accessories in the treatment room are also shown.

**Figure 2:**
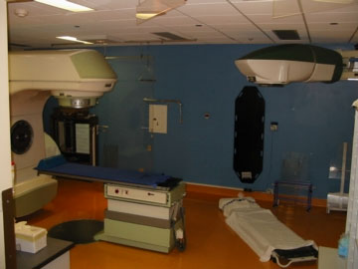
A photograph of the treatment room showing the location of the 10‐MV LINAC and the dedicated cobalt‐60 total body irradiator.

The cobalt unit irradiator was installed in the corner of the Clinac‐18 bunker, away from both the LINAC and the maze entrance, as shown in Fig. 1 (a schematic diagram of the layout) and in Fig. 2 (a photograph of the two machines). A two‐arm supporting bracket with a central attachment plate, designed specifically to hold the head‐and‐neck assembly, was constructed of heavy‐duty steel and anchored across the corner of the concrete bunker at a height of approximately 2.5 m from the floor. The head‐and‐neck assembly was placed in a specially constructed steel crib that had attachment points for hooks and chains. Heavy machine riggers raised the crib containing the head‐and‐neck assembly and positioned the coupling ring (located at the neck of the head‐and‐neck assembly) of the cobalt unit in the central attachment plate of the supporting bracket, where it was then fixed with bolts and stress shields. The steel crib is stored for future cobalt source changes.

**Figure 3:**
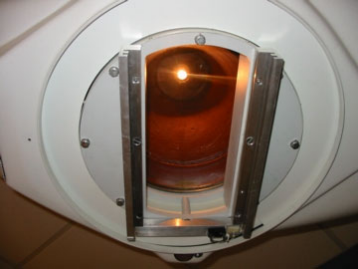
The custom‐made collimator, which replaces the conventional inter‐leaved depleted uranium collimator, is shown with the dual accessory rails. The field light located on the source rod can also be seen.

**Figure 4:**
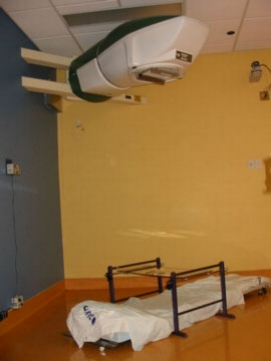
A photograph of the cobalt unit used to treat a patient in the prone/supine orientation. Anodized aluminum supports for positioning custom lung attenuators directly above the patient stretcher are also shown.

The intended purpose of the cobalt TBI irradiator is to produce a large fixed radiation field at an extended source‐to‐surface distance (SSD) for total‐body irradiation. To accomplish this, the conventional inter‐leaved depleted uranium collimator was removed, and a specially designed fixed‐field collimator was constructed out of overlapping hardened lead sandwiched between two sheets of steel to provide a rectangular field shape, as shown in Fig. 3. The collimator was fitted with a double accessory rail that was affixed to the existing attach points on the head‐and‐neck assembly. Custom acrylic trays were constructed for the accessory rails. The geometry of the final machine location provides a nominal source‐to‐floor distance of approximately 250 cm, with a fixed field size on the floor of approximately $1.3\times 2.7\;m^{2}$, as shown in Fig. 4.

The control cables for the cobalt irradiator were routed through the ceiling to a specially constructed junction box, and then to the original cobalt operator console. The junction box and the cobalt console are located in the same area as the LINAC console. Since the treatment room is a shared bunker, a selector switch ensures that only one of the two treatment machines (either the 10‐MV LINAC or the cobalt TBI unit) can be in operation at any given time. Existing safety switches used for the LINAC, such as the emergency off and last‐person‐out circuits, were also incorporated into the cobalt unit safety circuitry. Prior to initial use in the new configuration, the source drawer piston mechanism was tested by first operating it outside of the cobalt unit, while it was not attached to the radioactive source. The field light on/off switch was moved from the machine head to the wall, and transverse and sagittal ceiling‐mounted positioning lasers were installed to aid the placement of the patient into the treatment position.

## III. RESULTS AND DISCUSSION

Additional room shielding was not required for our 10‐MV bunker, since the cobalt‐60 beam is fixed in a manner pointed toward the floor, and the bunker is built on bedrock. Above the cobalt unit is a parking lot, and the bunker has a concrete ceiling thickness of 92 cm, a thickness more than adequate to shield for head leakage and room scatter produced by the cobalt unit. Measurements taken with a calibrated survey meter directly above the cobalt unit in a parking lot with the unit in the “beam‐on” position aimed toward the floor indicated exposure rates of about 0.1 μSv/h for a cobalt source having a nominal activity of about 100 TBq (2703 Ci).

### A. Off‐axis ratios

The large field size at the relatively short extended SSD produces an open‐beam dose profile that is unacceptable for clinical use. To improve the beam flatness at the treatment distance, a custom flattening filter, made of eleven 0.4‐mm lead sheets on the beam central axis, was manufactured to give a beam with a ±3% flatness over 190 cm in the inferior‐superior aspect by 80 cm in the transverse aspect at a depth of 10 cm in water.

**Figure 5:**
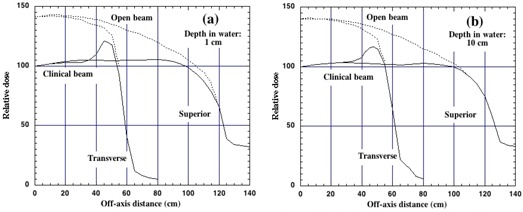
Off‐axis ratios are shown for the open beam (dashed curves) and the beam with the custom‐made lead filter (solid) as measured at a depth of 1.0 cm in (a) and 10 cm in (b) in water along the inferior‐superior and transverse aspects of the beam having a nominal field size on the floor of $1.3\times 2.7\;m^{2}$. Both figures are normalized to 1.0 at the central beam axis for the beam attenuated with the custom‐built flattening filter.

**Figure 6:**
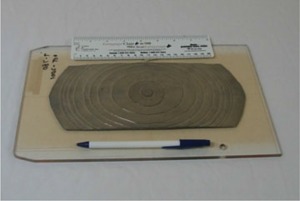
A photograph of the flattening filter made from 11 sheets of 0.4‐mm lead and affixed to a custom‐built acrylic support tray.

**Figure 7:**
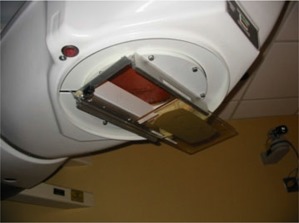
A photograph of the flattening filter partially inserted into the custom‐built acrylic support tray.

**Figure 8:**
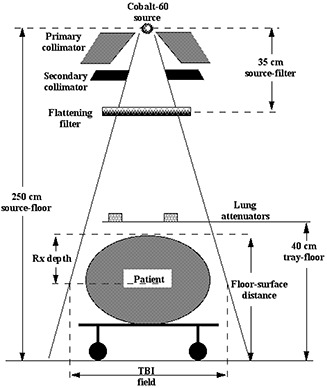
A schematic diagram of the treatment geometry showing the patient in cross‐section at the central axis lying on the stretcher. Typical distances are also indicated.

Figures 5(a) and 5(b) show the beam flatness measured at depths of 1.0 cm and 10.0 cm for an SSD of 220 cm with and without the custom‐made flattening filter in both the inferior‐superior and transverse beam axes. An SSD of 220 cm with a floor‐to‐surface distance of about 30 cm represents the clinical setup used to treat patients on a stretcher in the AP/PA orientation. The flattening filter is deliberately smaller than the treatment field in the transverse dimension and consequently produces a relatively steep rise of the off‐axis ratio outside of the clinically useful region of the beam. This permits viewing of the light field about the patient to help in patient setup and to provide an additional confirmation of the presence of the filter by staff prior to treatment. The flattening filter (shown in Fig. 6) is mounted on the custom‐built accessory rail (Fig. 7) and is locked in place with a set screw. Its presence is detected with a beam‐inhibiting interlock to ensure that clinical use of the machine does not occur without the flattening filter. The AP/PA treatment geometry is shown schematically in Fig. 8, where the transverse central axis aspect of the patient is shown on the treatment stretcher, and typical relevant setup distances are indicated. The standard prescription point at patient midseparation on the central axis is also shown.

### B. Percentage depth dose and tissue‐phantom ratio

**Figure 9:**
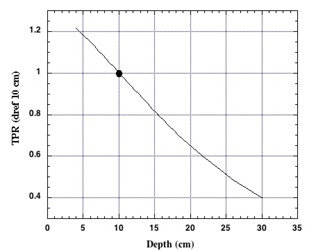
TPRs measured for the fixed field cobalt TBI irradiator with a $D_{\text{ref}}$ of 10 cm and a source‐$D_{\text{ref}}$ distance of 231 cm.

Both the percentage depth doses (PDDs) and tissue‐phantom ratios (TPRs) were measured over a range of clinically relevant distances from the source. The PDD and TPR are insensitive to relatively small changes in SSD caused by differences in patient separation experienced along the central axis of the beam. Patients having a central axis separation along the AP/PA aspect of less than 30 cm can expect to experience a dose gradient of up to 12% along the midline axis due to the inherent dose gradient. This dose gradient is somewhat worse than the gradient of 8% experienced with our previous LINAC‐based sweeping beam setup, although it is still clinically acceptable.^(^ ^3^ ^,^ ^4^ ^)^ The TPR curve with a reference depth $\left(D_{\text{ref}}\right)$ of 10 cm measured with a typical source‐to‐reference point distance of 230 cm is shown in Fig. 9.

### C. Surface dose and buildup region

**Figure 10:**
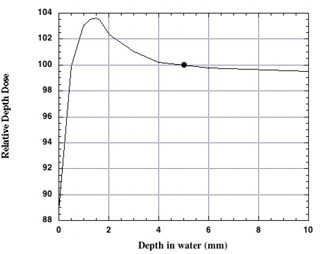
Surface dose at an SSD of 230 cm measured through the first few millimeters for the total body irradiation beam with the flattening filter in place.

The surface dose and the dose buildup region for a typical patient TBI setup were measured with a parallel‐plate Attix chamber having a window thickness of $4.8\;\text{mg}/{\text{cm}}^{2}$.^(5)^ Results, when normalized to 100% at a depth of 5 mm, are shown in Fig. 10. The surface dose was 89%, with a $z_{\max}$ depth of 1.5 mm and a PDD of 103.6% at $z_{\max}$. Similar results were seen for the extended SSD beam without the custom flattening filter. This buildup data for large fields and extended SSD for cobalt beams is similar to that reported by other authors,^(^ ^6^ ^,^ ^7^ ^)^ and the contaminated surface dose is in fact an advantage for TBI treatments because it alleviates the need for skin bolus. Percent depth dose at depths of 5 cm, 10 cm, and 20 cm was found to be 87.8%, 71.4%, and 43.6%, respectively. This data may be compared to the classic cobalt data for a $10\times 10\;{\text{cm}}^{2}$ field at an SSD of 80 cm, where the PDDs at depths of 5 cm, 10 cm, and 20 cm are 78.8%, 56.4%, and 27.4%, respectively, or for a $45\times 45\;{\text{cm}}^{2}$ field at an SSD of 80 cm, where the PDDs at depths of 5 cm, 10 cm, and 20 cm are 82.9%, 64.1%, and 36.8%, respectively.^(8)^

### D. Shutter error and absolute output

The shutter error was determined to be 0.02 min, a value identical to that in effect when the unit was in clinical use as a conventional isocentric cobalt teletherapy unit. The absolute output was determined using the TG‐51 protocol of the American Association of Physicists in Medicine (AAPM), a water tank, and a waterproof Exradin (model A12) ionization chamber having a cobalt‐60 water calibration coefficient, $N_{D,w}^{\text{Co}‐60}$ traceable to a standards laboratory. The waterproof Exradin chamber was placed at a reference depth of 10 cm in a water tank at a source‐to‐chamber distance of 230 cm, a clinically relevant distance in our treatment setup.

### E. Treatment setup

During each treatment, half the midline dose is delivered with the patient in the supine position and the other half with the patient in the prone position (AP and PA). This AP/PA treatment regime minimizes the central axis and off‐axis patient separation, and makes the treatment of pediatric patients and patients requiring partial kidney and liver shielding easier. Patients are treated with lateral bolus to ensure full side‐scattering conditions. The treatment stretcher is at a fixed distance from the floor, and a TPR‐type setup is used for the calculation of the beam‐on time.

As with our previous sweeping beam TBI technique, we use lead attenuators to match the lung dose as close as possible to the prescribed dose. Our experiments with thermoluminescent dosimeters (TLDs) in heterogeneous phantoms simulating patients’ lungs have shown that a lead attenuator thickness of 2.8 mm brings the lung dose in a humanoid phantom to within ±5% of the prescribed dose. Without the lead lung attenuators, the lung dose exceeds the prescribed dose by up to 18%.

For every patient the shape of the 2.8‐mm‐thick lung attenuator is determined by using portal films (PPL, Kodak, Rochester, NY) produced with the cobalt beam at the time of the initial treatment planning. The lung attenuators are supported on a removable Lucite tray typically 10 cm above the patient's surface. Approximately 15% of patients also require partial shielding to achieve a lower‐than‐prescribed dose to organs such as the kidneys and liver. The dose under these partial shields is typically between 40% to 80% of the prescription dose, depending upon the physician's prescription. The shields are custom‐designed using CT planning and placed onto the removable Lucite tray, which also holds the lung attenuators. These partial organ shields are constructed with a low‐melting alloy (Cerrobend), which has a half‐value thickness of about 20 mm in our broad beam clinical setup. Placement of organ shields is verified by the use of verification films (XV‐2, Kodak, Rochester, NY) placed on the floor under the patient stretcher during treatment.

Treatment time for a typical adult is of the order of 20 min to deliver 100 cGy to midplane on central axis from one direction with a source having a nominal activity of 100 TBq (2703 Ci). Treatment regimens vary from one fraction of 200 cGy to the more standard dose regime of 1200 cGy given in either six or eight fractions delivered in two treatments per day.

The fixed‐field cobalt TBI technique was verified using TLDs in a humanoid phantom that was irradiated under conditions similar to the treatment setup. Differences between measured and expected doses were typically less than ±5%. The technique is also checked on an annual basis whereby parameters such as field flatness and symmetry, PDD and TPR, beam output, as well as shutter error are verified. In addition, our technique is periodically monitored by the Radiological Physics Center using the mail‐in TLD program and site visit audits.

### F. Emergency procedures

The unusual geometric setup of this treatment unit at 2.5 m from the floor means that easy access to the source drawer in the event of an emergency is not possible. Our emergency procedure for this unit is in fact similar to that of our conventional isocentric SAD 80 cm cobalt unit. In the event that the source fails to return to its safe position, staff are instructed to (1) remove the patient while avoiding the primary beam as much as possible, (2) leave the room and lock the door, and (3) call physics personnel for assistance. The patient is positioned on a floor stretcher equipped with casters so that moving the patient is relatively easy. Thus, moving the patient during an emergency situation does not require that personnel enter the primary beam. The limits of the primary beam are marked on the floor to further aid staff in determining appropriate positioning during an emergency procedure.

Prior to implementing the TBI technique clinically, equivalent dose rates at various locations outside of the primary beam, with a humanoid‐like phantom in the treatment position, were measured. At distances of 0.3 m, 0.6 m, and 1.0 m from the beam edge, equivalent dose rates were found to be approximately 15, 4, and 2.5 mSv/min, respectively. During emergency practice sessions with personnel, it has been observed that 15 s seems to be more than adequate to remove the patient from the treatment position and leave the room, thus indicating that there would be little risk associated with removing the patient during emergency conditions. So far, an emergency situation has never been encountered. In addition to the established and regularly rehearsed emergency procedures, license conditions require us to have in the treatment room at all times a “Tee‐bar,” which is available to help service personnel in returning the stuck source to the safe position. A dedicated stepladder is always located in the treatment room to expedite access to the cobalt unit during emergency service. As part of regularly scheduled safety training, service personnel and physicists receive dedicated emergency procedure instructions regarding this treatment unit.

### G. Chamber constancy verification

**Figure 11:**
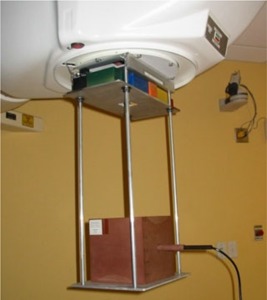
A photograph of the jig used to hold a block of solid water at a fixed SSD used in determination of response constancy of ionization chambers. An Exradin chamber with a solid water sleeve is inserted into the block. The $10\times 10\;{\text{cm}}^{2}$ field size is achieved with the use of colored, free lead blocks, arranged on a plate at the level of the custom collimator.

In addition to clinical use, the installation of the cobalt unit in this configuration has allowed us to perform our regularly scheduled ionization chamber constancy measurements. It also allows us to transfer the cobalt chamber calibration coefficients $N_{D,w}^{\text{Co}‐60}$ from our secondary standard to our tertiary (field) calibration ionization chambers. A specially constructed jig (Fig. 11), which attaches to the accessory rails, holds a solid water phantom cube $\left(20\times 20\times 20\;{\text{cm}}^{3}\right)$ at a constant SSD of about 80 cm from the source in a fixed and reproducible geometry. This jig also permits the collimation of the large whole‐body field size to a $10\times 10\;{\text{cm}}^{2}$ beam at the surface of the water phantom cube with the use of a number of free lead shielding blocks, which are visible in Fig. 11 near the accessory rails. The solid water transfer phantom has a hole drilled at a depth of 10 cm to accept the ionization chamber in its solid water sleeve. Constancy measurements are carried out without the custom flattening filter in place, and a bypass circuit enables beam production under these special circumstances. This bypass circuit has an automatic reset at power down so that the normal interlock condition will inhibit the beam and alert the operator to the absence of the flattening filter prior to patient treatment.

## IV. CONCLUSIONS

Our stationary, wall‐mounted, fixed‐field cobalt technique has been in successful operation since November 2001, and over 130 patients have been treated with total body irradiation to date. Current treatment regimes vary from single fraction treatments of 200 cGy to multiple fraction treatments lasting three to four days and delivering up to 1200 cGy to the patient midplane. While the current trend in radiotherapy is to replace cobalt units with more versatile and technologically advanced linear accelerators, we believe that there remain many useful applications for cobalt units and that these units may conveniently share bunker space with an existing LINAC installation without requiring excessive departmental refurbishing.
